# Developmental Reaction Norms for Water Stressed Seedlings of Succulent Cacti

**DOI:** 10.1371/journal.pone.0033936

**Published:** 2012-03-30

**Authors:** Ulises Rosas, Royce W. Zhou, Guillermo Castillo, Margarita Collazo-Ortega

**Affiliations:** 1 Center for Genomics and Systems Biology, New York University, New York City, New York, United States of America; 2 Instituto de Ecología, Universidad Nacional Autónoma de México, México Distrito Federal, Mexico; 3 Facultad de Ciencias, Universidad Nacional Autónoma de México, México Distrito Federal, Mexico; Lawrence Berkeley National Laboratory, United States of America

## Abstract

Succulent cacti are remarkable plants with capabilities to withstand long periods of drought. However, their adult success is contingent on the early seedling stages, when plants are highly susceptible to the environment. To better understand their early coping strategies in a challenging environment, two developmental aspects (anatomy and morphology) in *Polaskia chichipe* and *Echinocactus platyacanthus* were studied in the context of developmental reaction norms under drought conditions. The morphology was evaluated using landmark based morphometrics and Principal Component Analysis, which gave three main trends of the variation in each species. The anatomy was quantified as number and area of xylem vessels. The quantitative relationship between morphology and anatomy in early stages of development, as a response to drought was revealed in these two species. Qualitatively, collapsible cells and collapsible parenchyma tissue were observed in seedlings of both species, more often in those subjected to water stress. These tissues were located inside the epidermis, resembling a web of collapsible-cell groups surrounding turgid cells, vascular bundles, and spanned across the pith. Occasionally the groups formed a continuum stretching from the epidermis towards the vasculature. Integrating the morphology and the anatomy in a developmental context as a response to environmental conditions provides a better understanding of the organism's dynamics, adaptation, and plasticity.

## Introduction

The phenotype is the result of complex instructions and interactions specified by the genotype, in the context of environmental conditions. However, the phenotype is not a static output, but rather a dynamic product of the genotype-environment interactions during development. Characterizing phenotypic changes during development is important in developmental and evolutionary biology to help better understand phenotypic constraints and trade-offs. It is experimentally difficult to recognize phenotypic features potentially involved in such compromises, and the relevance of these changes during development. Moreover, characterization of developmental phenotypes at several levels of complexity might not provide enough information to infer putative trade-offs. Thus, it is important to characterize the different physical features of the phenotype during development, and also its phenotypic plasticity as a result of challenging environmental conditions that affect the success of the organism. This complexity of developmental responses to an environmental condition has been defined as Developmental Reaction Norms or DRN [1], [2]. Every trait could be assumed to have different DRN from one another if the traits are completely independent. In reality, many traits are somehow interrelated, and it is important to differentiate how each one contributes to the final phenotypic outcome. Thus, it would be ideal to build a quantitative framework to analyze several stages of the phenotype during development, as well as responses to an environmental condition. Here, the quantitative DRN for two complex interrelated traits were analyzed. This was done by measuring the morphology and the anatomy of two species of cacti seedlings, taking advantage of their slow growth and development.

Most cacti are succulent plants adapted to conditions with limited water availability. This feature confers cacti with a greater capacity to retain water that allows them to withstand severe and prolonged drought periods while maintaining photosynthetic rates. Water is mainly stored in the water-storage parenchyma of the cortex within the stems. Cacti have a small surface-volume ratio which allows them to store a maximum of water but with a minimum of transpiration area. Also, the root system extends horizontally and vertically with fine root hairs that develop during rainfall but die during drought to optimize the balance between water intake and loss. Stems have thick cuticles and often have trichomes and spines mainly at the apex to protect the apical meristem from solar radiation and heat damage [3], [4], [5]. In this experiment, two cacti species—*Polaskia chichipe* and *Echinocactus platyacanthus—*were analyzed during their early developmental seedling stages. These two species represent two of the most conspicuous life forms in succulent cacti, columnar and barrel, respectively.

*P. chichipe* (Goss) Backeberg is a columnar arborescent cacti with the following characters: 3–5 m in height, and profusely branched; the stems have 9–12 ribs; fruits are red and spherical, 20×20 mm, 6.8 g by weight [6]; seeds are black and 1.3 mm long [7]; distributed in Puebla and Oaxaca, Mexico [8]; flowering occurs between February and July. *E. platyacanthus* Link & Otto has a barrel stem shape with many ribs, adults plants are 50 cm to 2 m in height and 40 to 100 cm in diameter; the apex is sunken with abundant yellow wool [8]; fruits are dark brown, largely oblong to kidney-shaped, 35×14 mm, 3.5 g by weight; seeds are black with a smooth seed coat. It is an endemic species of Mexico, distributed in Coahuila, Guanajuato, Hidalgo, Nuevo León, Oaxaca, Puebla, Querétaro, San Luis Potosí, and Tamaulipas y Zacatecas, flowering between June and September [6].

There have been significant advances in understanding the early vegetative growth of succulent cacti, including physiological events such as germination [9], [10], [11], [12], morphological and anatomical features [13], [14], as well as metabolic status during development [15]. More recently, there has been a growing interest in understanding these developmental transitions in an eco-physiological context by exposing the organisms to a simulated or real challenging environment, such as drought [16], [17], [18], [19], [20]. Despite these advances, quantitative frameworks are still needed to compare early changes in cacti morphology and assess whether they are explained by quantitative anatomical changes when the plants are being challenged. In this paper, a quantitative framework is presented to show that changes in the morphology are explained by changes in anatomical features in the seedlings of two cactus species (*P. chichipe* and *E. platyacanthus*). These developmental changes are shown in the context of DRN as seedlings were grown under challenging drought.

## Results

**Figure 1:**
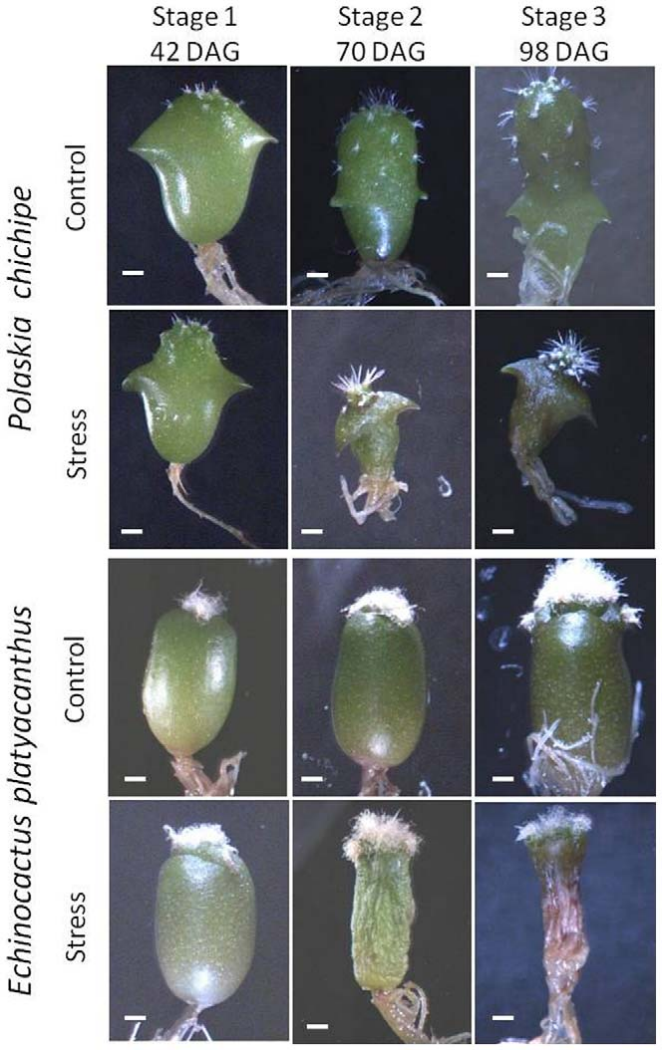
Seedlings of *Polaskia chichipe* and *Echinocactus platyacanthus* at three developmental stages and two water availability conditions. DAG: days after germination. Scale bar 1 mm for all images.

**Figure 2:**
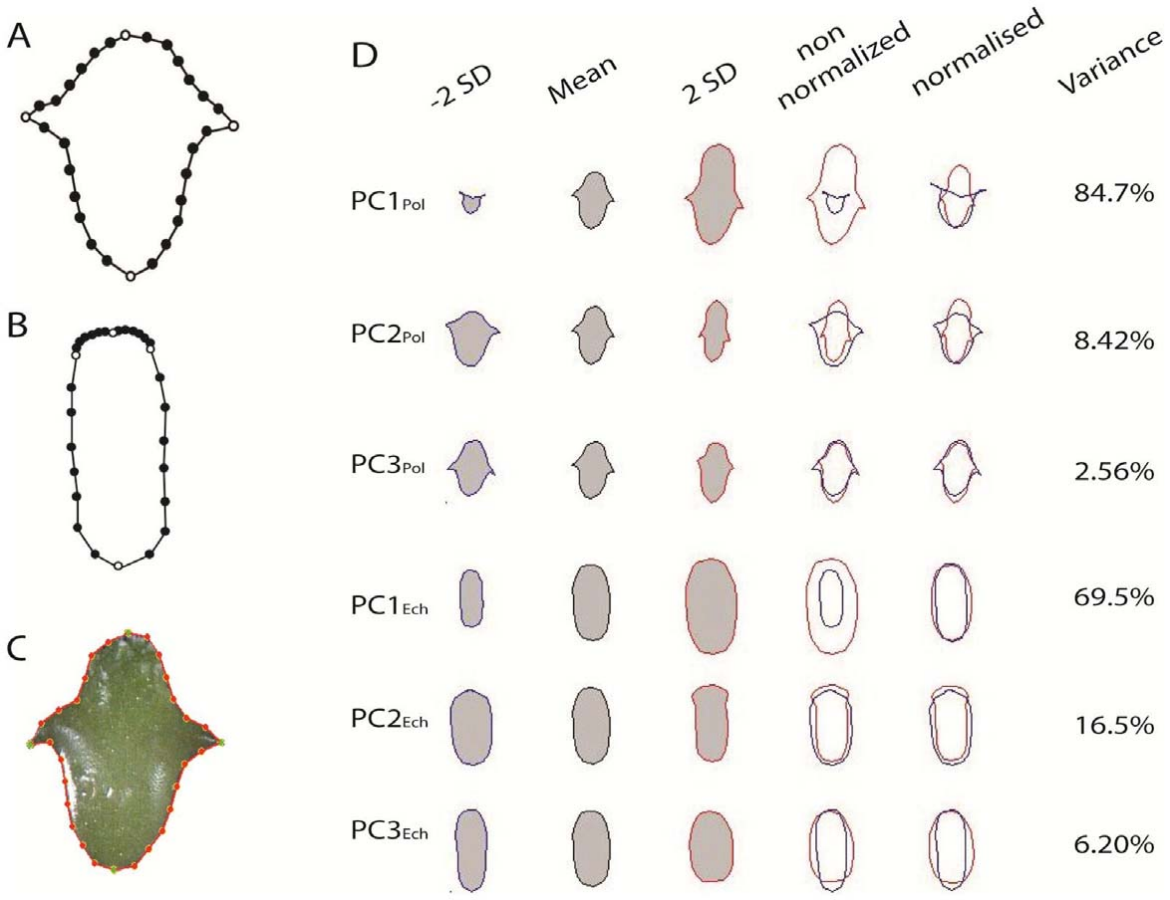
Morphometric analysis of seedling shape and size. **A,B**: 30-point template to capture the shape of both *Polaskia chichipe* and *Echinocactus platyacanthus* seedlings. Open circles correspond to primary landmarks which are placed on recognizable features of the seedlings (base, cotyledonary areoles, and apex); filled circles correspond to secondary landmarks evenly spaced between primary landmarks. **C**: Example of the 30-point model template fitted onto a photographed seedling. **D**: Principal Component Analysis of *Polaskia chichipe* (PCA_Pol_) and *Echinocactus platyacanthus* (PCA_Ech_) seedlings. Mean shapes with and without procrustes for size are shown. SD: Standard deviation. PC1_Pol_ shows elongation of the apex with size effects.

An *in vitro* system was implemented to study the anatomical and morphological DRN of *P. chichipe* and *E. platyacanthus* under a well-watered condition and a challenging drought condition (manitol added), sampled at three developmental stages (Figure 1). Every stage and treatment had 6 replicates in *P. chichipe* and 9 replicates in *E. platyacanthus*. To capture the morphology, side views of the seedlings were obtained, and a collection of 30 corresponding landmarks were placed to represent the outline of each digital image (Figure 2A–2C). This approach was taken because shape and size can be simultaneously obtained, as opposed to traditional measurements of size (i.e. length or width) or shape (i.e. ratio length-width) on their own. The landmark data was aligned according to their centroid, and rotated to minimize the variation between corresponding landmarks (procrustes for translation and rotation). The transformed landmark data was then used to identify the main features of shape and size variation using Principal Component Analyses (PCA), from which morphologies can be quantified as Principal Components (PCs). Detailed descriptions of the procedures can be found in Langlade et al (2005) and Bensmihen et al (2008) [21], [22].

The datasets of *P. chichipe* and *E. platyacanthus* gave three PCs describing more than 90% of the variation in shape and size in both species (Figure 2D). The rest of the PCs were ignored as they captured less than 2.5% of the variation in both species. Despite having different morphologies, the features captured by PCA_Pol_ and PCA_Ech_ are comparable. PC1_Pol_ and PC1_Ech_ mainly capture the size and turgence variation between seedlings; however PC1_Pol_ also captures the elongation of the apex, while PC2_Pol_ and PC2_Ech_ capture the elongation of the apex, regardless of the plant size. PC3_Ech_ seems to capture the turgency variation of the hypocotyls, whereas PC3_Pol_ captures a more subtle variation of the hypocotyl shape.

**Figure 3:**
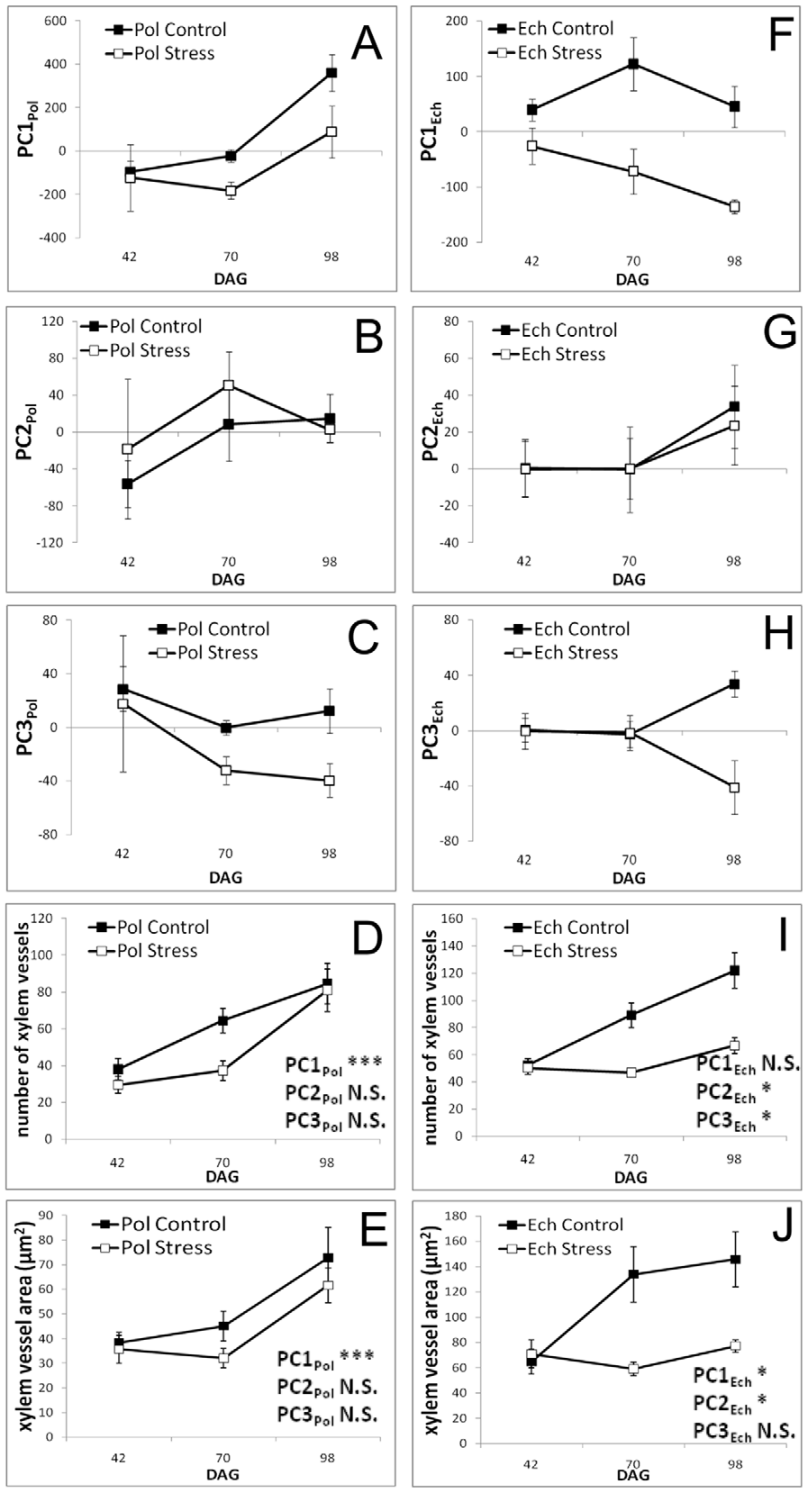
Quantification of the morphology and the anatomy in cacti seedlings. **A–C**: The morphology of *Polaskia chichipe* expressed as PC_Pol_. **D–E**: The number and area of xylem vessels in *Polaskia chichipe*. **F–H**: The morphology of *Echinocactus platyacanthus* represented as PC_Ech_. **I–J**: The number and area of vessels in *Echinocactus platyacanthus*. **D–E**,**I–J**: Significant PCs from the regression *y_i_* = β_PC1_+β_PC2_+β_PC3_+ε_i_ are highlighted. * p<0.5, *** p<0.0005. Error bars represent standard error. DAG: days after germination.

The morphological effects of seedling age and the water availability response can be represented according to their PC quantification. In *P. chichipe*, PC1_Pol_ shows that the size, turgency, and elongation of the apex increases with age in Control conditions, but the stress treatment reduced the increment (Figure 3A); PC2_Pol_ shows that there is no significant elongation of the apex due to the age of the seedling or the treatment (Figure 3B); PC3_Pol_ shows that the shape of the hypocotyl does not vary according to the age of the seedling, but is modified by the stress treatment (Figure 3C). In *E. platyacanthus*, PC1_Ech_ shows a reduction in seedling size and turgency due to the stress treatment, but not age (Figure 3F); PC2_Ech_ shows no variation on the elongation of the apex due to age or treatment (Figure 3G); PC3_Ech_ shows a highly significant increase in the turgency of the hypocotyl due to age, which is stunted in the water stress treatment (Figure 3H). The results show that quantitative morphological changes due to age and water treatment are not comparable in these two species.

### Morphological variation is associated to quantitative anatomical features

To test whether morphological changes in shape and size are related to anatomical features, semi-fine sections of the shoot were obtained. The number of xylem vessels was counted, and the xylem vessel area was quantified. In *P. chichipe* the number of vessels increases with age, even though it was initially reduced by the stress treatment and recovers in the later stage (Figure 3D); when adding up all the vessel areas, there was an increment towards the last developmental stage, but the stress treatment shows no significant effect (Figure 3E). In *E. platyacanthus*, both the number of xylem vessels and the total vessel area increase with age, and both are stunted by the stress treatment (Figure 3I–3J). In the seedling stage, the parenchyma cells used for water storage are very similar between these two species (Figures S1, S2). These results show that morphological quantitative changes might be the result of different anatomical early responses of seedlings of these species.

To test the quantitative relation between morphology and anatomy, multiple regression analyses were done on the number of xylem vessels and the xylem vessel area, as a response of PC1, PC2, and PC3 (i.e. *y_i_* = β_PC1_+β_PC2_+β_PC3_+ε_i_). Both the variation in the number and area of xylem vessels in *P. chichipe* were explained by PC1_Pol_ (stdβ_number_ = 0.70, *p_number_*<0.0001; stdβ_area_ = 0.64, *p_area_* = 0.0002;), the size, turgency, and elongation of the seedling (Figure 3D–3E). In contrast, in *E. platyacanthus* the variation in xylem vessel number was explained by PC2_Ech_ (stdβ_number_ = 0.36; *p_number_* = 0.008) and PC3_Ech_ (stdβ_number_ = 0.29; *p_number_* = 0.042), the elongation of the apex and the turgency of the hypocotyls. Meanwhile, the area of xylem vessels was explained by PC1_Ech_ (stdβ_area_ = 0.33; *p_area_* = 0.024) and PC2_Ech_ (stdβ_area_ = 0.29; *p_area_* = 0.040), the size and turgency of the seedling as well as the elongation of the apex (Figure 3I–3J). Overall, these analyses show connections between the DRN of anatomical and morphological features of the seedlings under water stress conditions as well as diversification of stress coping strategies.

## Discussion

DRN in response to water stress were obtained using seedlings of *P. chichipe* and *E. platyacanthus*. The complexity of the phenotypes was analyzed in terms of morphology and anatomy. The morphology was evaluated using landmark based morphometrics, which resulted in three main trends of the variation (PC1, PC2, and PC3) in each species, PCA_Pol_ and PCA_Ech_. The anatomy was quantified as the number and the area of xylem vessels. Quantitative features of the morphological variation throughout development were found to be associated with vascular anatomical changes. The quantitative analysis of morphology and anatomy showed that DRN in response to water stress follow different trajectories in these two cacti species, and that morphological responses are correlated to anatomical changes.

DRN have been argued to be the result of mechanisms of adaptation to cope with variations in the environment [2], [23]. Thus, the ontogeny might be reflecting adaptive developmental trajectories toward the adult stage and life form of the organism. For example, in cacti, it has been found that seed germination responses to light and temperature are associated with the adult life form (column or barrel cacti), and interpreted as an ecological adaptation to their corresponding niches [10], [11]. *P. chichipe* corresponds to the column cactus category, whereas *E. platyacanthus* is a barrel cactus. The adult plant in *P. chichipe* can reach 3–5 m in height, and the terminal branches can have 0.1 m in diameter; on the other hand *E. platyacanthus* has 0.5–2 m in height, and 0.4–0.8 m in diameter [6]. It is possible that the morphological and anatomical diversification in DRN of these two species is the result of more complex ecological adaptations of cacti life forms, which might involve germination as well as developmental characters.

The anatomy of succulent cacti during development is considered to be relatively simple, consisting of vascular bundles surrounded by large regions of parenchyma, and thick epidermal layers [24]. This makes succulent cacti an ideal model to study water stress responses at the anatomical level. For instance, the vasculature shows consistent xylem vessel length throughout development which is a feature of primary growth retained from juvenile characters; thus, the adult could be considered a giant seedling [25]. This implies that longitudinal anatomical changes are not as important as transversal anatomical changes. Hence, in this work the transversal change on the vasculature of seedlings was the primary focus. These changes turned out to be significant during development, and affected by the water stress treatment. In the root, it has been shown that drought affects their conductivity and anatomy [26]. Those changes in vasculature that occur during early seedling development are affected by water stress, and possibly have an effect on water conductivity in the shoot.

### Qualitative anatomical adaptations in adult plants are also seen in seedlings

**Figure 4:**
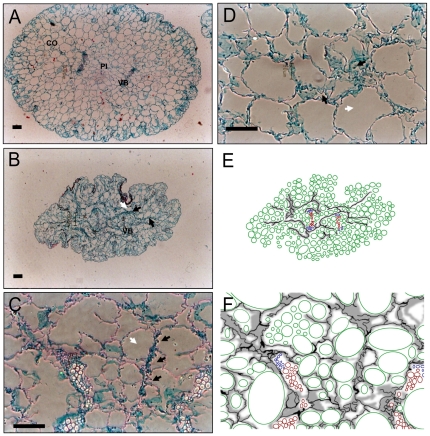
Histological sections and schematic representations of *Echinocactus platycanthus* hypocotyls. Transversal sections were obtained 3–4 mm above the base of the shoot. **A**: Seedling of a Control treatment. **B** Seedling of a Stress treatment. **C** Vascular bundle area showing details of the collapsible cells and areas of collapsible tissue. **D**: Section of parenchyma showing turgid cells next to collapsed cells. **E–F**: Schematic representation of B,C respectively: turgid cells in green, collapsed cells in grey, xylem cells in red, and phloem cells in blue. **A–B**: Bright field microscopy; **C–D**: Phase contrast microscopy. White arrows show a turgid cell, black arrows show groups of collapsible cells. Scale bar 100 µm. CO: cortex; PI: pith; VB: vascular bundle.

In adult cacti, most of the tissue is constituted by water storage parenchyma with cellular spaces that are mostly occupied by the vacuole. In some parenchyma regions, however, turgid cells are adjacent to collapsed cells, which have been named the collapsible tissue [27], [28]. This is because the cell walls in the collapsible tissue have properties that allow the cells to expand and shrink to store water, presumably as a response to the water availability conditions. This phenomenon has been reported in seedlings of another cactus [18], and was observed in seedlings of *P. chichipe* and *E. platyacanthus*. In the seedling stage, the parenchyma cells are very similar between these two species, thus for simplicity only the latter is shown. The collapsible tissue was observed more often in seedlings that were subjected to the water stress treatment (Figures 4A and 4B). Groups of collapsed cells were located inside the epidermis, resembling webs surrounding turgid cells (Figure 4B and 4E), around the vascular bundles, spanning across the pith (Figure 4C and 4F) and in the cortex (Figure 4D).

Quantitative changes in the morphology of *P. chichipe* and *E. platyacanthus* seedlings were associated with features of the vasculature, and therefore the hydraulic status. Moreover, the qualitative feature of collapsible cells in the parenchyma tissue described by Mauseth [27] in adult plants was also observed in this experiment, mostly in water stressed seedlings. This is consistent with previous findings of older seedlings of another cactus species [18]. Interestingly, the groups of collapsible tissue were often surrounding the vascular bundles, occasionally making a continuum between the vasculature and the epidermis. This phenomenon has been explained in adult cacti as an adaptation to maintain meristematic tissues and vasculature functionality [27], or water exchange balance between water-storage parenchyma and chlorenchyma that maintains the osmotic pressure on tissues to sustain photosynthetic metabolism [19]. In seedlings, most of the cortex is presumably photosynthetic, and therefore the collapsible tissue is unlikely to be important to maintain photosynthetic activity. Yet, it is not clear how the collapsible tissue might play an adaptive role during seedling establishment and development and its complex interplay with other mature tissue dynamics i.e. chlorenchyma-water storage parenchyma [29], or wooden tissues [30]. The development of new quantification methods to evaluate the collapsible tissue and the web-like structure of collapsible cells will be important to understand their adaptive significance.

The xylem is a complex tissue that both functions as a means of transport for water and solutes, as well as structural support. In adult cacti, this tissue has been known to play a main role in plant support, and affects the shape and the biomechanical properties of the plant [31]. Qualitative comparisons between seedlings and adult plants have been discussed to be associated to strategies, growth, and morphologies across development [15]. Other works have examined the type of xylem cells related to the morphologies of life forms in the subfamily Cactoideae [5]. The experiment aimed to show a quantitative relationship between the anatomical features and the morphology of the plant in early developmental stages, during which its shape is highly susceptible to changes in water availability. For this, assumptions were required (sections of the hypocotyls and shapes of 2D images) as the anatomy and morphology is complex; thus it is likely that other parts of the plant will show different morphology-anatomy relations. Future work is required to further characterize and quantify the structural anatomy and morphology of cacti in a more comprehensive way [32].

The implementation of novel morphometric methods (i.e. [21], [22], [33], [34]) to assess the features of organs and organisms allows a quantitative comparison of morphology and anatomy. The importance of integrating these two aspects of the organism has been highlighted as one of the new syntheses in biology [35]. Integrating morphology and anatomy in a developmental context, as a response to environmental conditions (DRN; [2]), will provide a better understanding on the organism dynamics, adaptation, life history, and plasticity.

## Methods

### Biological material

*Polaskia chichipe* seeds were collected from a population located at 17°45′N, 97°44′W, and *Echinocactus platyachantus* seeds from a population at 18°24′N, 97°26′W. Both locations belong to the Tehuacán-Cuicatlán Valley in Mexico. Seed material was collected in May–June of 2001 with permission of Secretaría de Medio Ambiente y Resursos Naturales (Mexico), permit No.DOO.02-1139 (no field experiments were done during this study). Prior to germination, seeds were treated with sulfuric acid for 15 s, and disinfected with solutions of Tween80 (30 mins), ethanol 70% (2 mins), and bleach 20% (15 mins), plus three rinses with sterilized water. Seeds germinated in MS medium 50%, 1% agar. Germination of these two species spanned between 20–30 days [36]; thus germination was recorded daily to determine seedling age. At 35 days after germination (DAG), seedlings were transferred to liquid MS 50% medium for the control treatment, and with Manitol added (101.76 g L^−1^) for the stress treatment. Germination and water stress treatments were done in a controlled environment chamber at 25°C±1, photoperiod 16/8 h, and light intensity 11960–15640 µmol m^−2^ s^−1^. Seedlings were photographed at 42, 70, and 98 DAG using a stereoscopic microscope (Zeiss). Digital photographs were taken at magnifications 2× and 2.5×. The images were re-scaled to 72 ppi, to account for the magnification that was used.

### Analysis of anatomy

Seedlings were fixed in FAA (Formol, acetic acid, ethanol; 2∶1∶10). Transversal sections of 8–14 µm in paraplast were obtained from the shoot, 3.5 mm above the base of the seedling. Sections were stained with safranin-fast green to distinguish the xylem vessels from the rest of the bundle cells. Photographs were taken with a microscope (Olympus), number of vessels was counted, and the area of each vessel was measured using the software Zeiss Image 3.0.

### Quantification of morphology and statistical analyses

The complex morphology of cacti seedlings has been extensively described qualitatively [13], [14], but there are few studies that have aimed to quantify early morphological changes [18]. Landmark based morphometrics have been shown to provide robust frameworks for the quantification of organ shape in model systems [21], [22], [34]. To simplify the three-dimensional shape of the seedling, we assumed that the side view of the shoot is sufficient to describe the morphology (Figure 1). The *Polaskia chichipe* plants were aligned so that the two cotyledonary areoles pointed to the side when the photos were taken. A template of 30 landmarks was created to capture the main shape features. The landmarks were classified as either primary for those that correspond to identifiable features of the seedling, or secondary for those evenly spaced between primary landmarks. The 30 point landmark template had four primary landmarks: one at the apex, one at the base, and one at each of the cotyledonary areoles. There were 26 secondary landmarks: 6 distributed between each of the cotyledonary areoles to the apex (12 in total), and 7 between each cotyledonary areoles and the base of the seedling (14 in total). Using the point datasets, shapes were preprocessed to align them and rotate them to minimize the variation between corresponding landmarks (procrustes for translation and rotation). All morphometric processing and analyses were done using the software “Shape Model Toolbox.” [21], [22] Thus, Principal Components for shape and size were calculated for every dataset. Regression analyses of the PCs were done using the statistical software JMP®Genomics 5.1 (SAS Institute Inc., USA). Every histological section was pair-wise matched to the corresponding morphological image and PCA data points.

## Supporting Information

Figure S1 Sections of the time-course of development in *Polaskia chichipe*. DAG: days after germination. CO: cortex; PI: pith; VB: vascular bundle; TC: turgid cells; GCC: groups of collapsible cells. Magnifications 2.5×.
(TIF)

Figure S2 Sections of the time-course of development in *Echinocactus platyacanthus*. DAG: days after gemination. CO: cortex; PI: pith; VB: vascular bundle; TC: turgid cells; GCC: groups of collapsible cells. Magnifications 2.5×.
(TIF)
