# Supplementary figures and images for: Developmental Reaction Norms for Water Stressed Seedlings of Succulent Cacti
Source: PLoS One. 2012 Mar 30;7(3):e33936. doi: 10.1371/journal.pone.0033936 (PMC3316504; doi:10.1371/journal.pone.0033936)

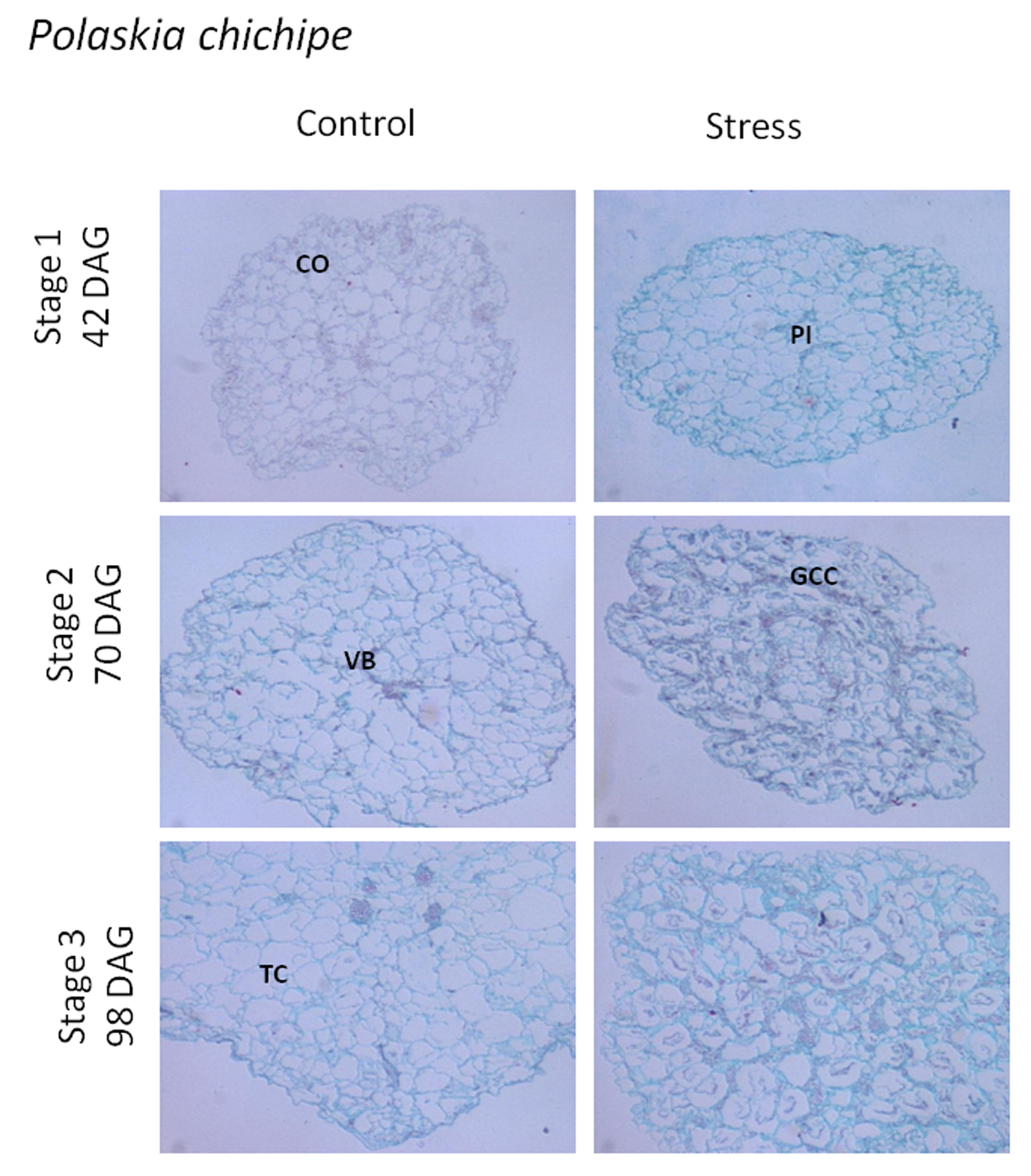

Supplement: Figure S1 — Sections of the time-course of development in Polaskia chichipe. DAG: days after germination. CO: cortex; PI: pith; VB: vascular bundle; TC: turgid cells; GCC: groups of collapsible cells. Magnifications 2.5×. (TIF) [file pone.0033936.s001.tif]

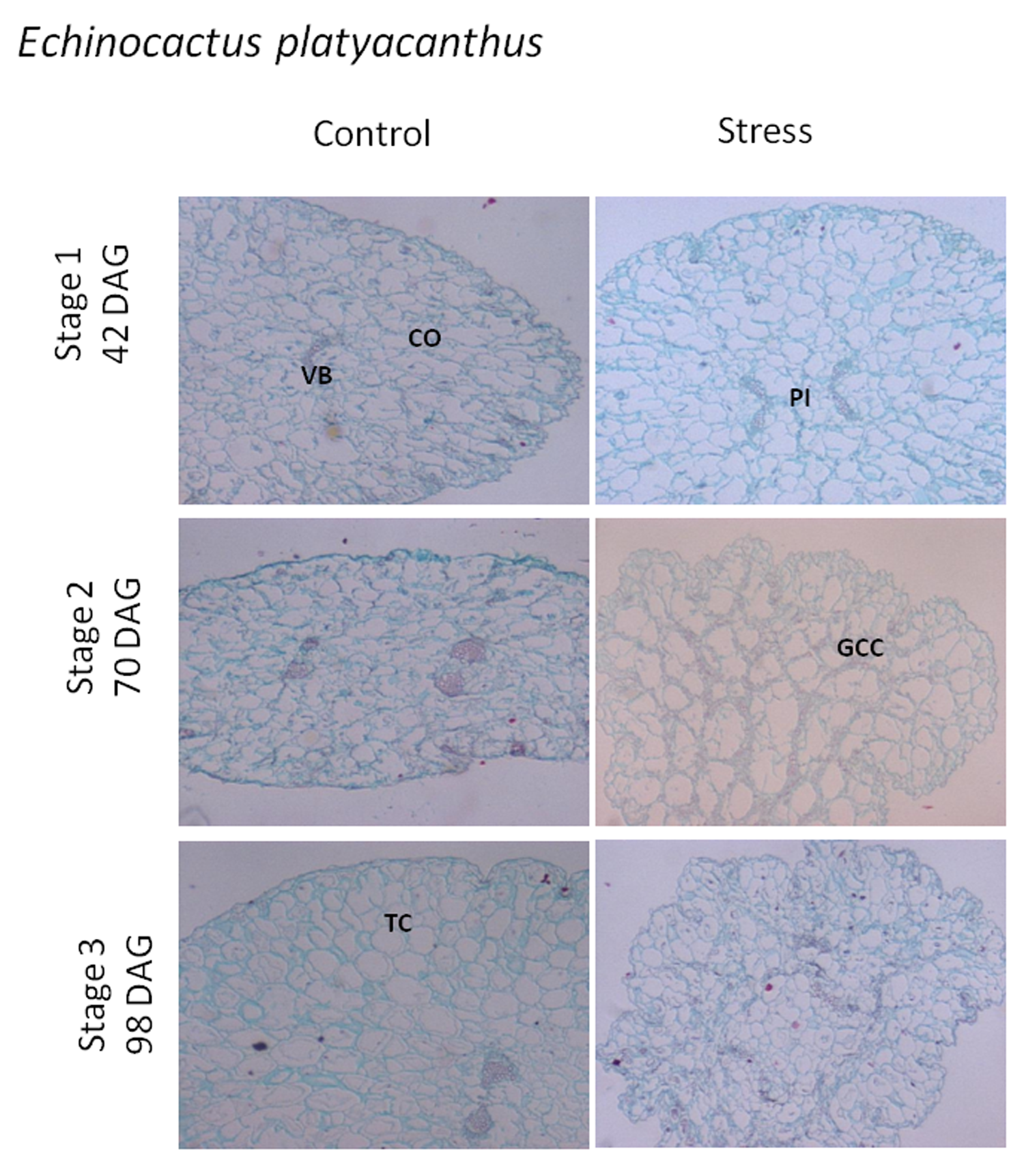

Supplement: Figure S2 — Sections of the time-course of development in Echinocactus platyacanthus. DAG: days after gemination. CO: cortex; PI: pith; VB: vascular bundle; TC: turgid cells; GCC: groups of collapsible cells. Magnifications 2.5×. (TIF) [file pone.0033936.s002.tif]
